# Mechanistic research approaches in music therapy for pain: Humanizing and contextualized options for clinician-researchers

**DOI:** 10.3389/fpain.2022.1002819

**Published:** 2022-10-04

**Authors:** Andrea McGraw Hunt

**Affiliations:** Department of Music, Music Therapy Program, Rowan University, Glassboro, NJ, United States

**Keywords:** music medicine, music therapy, music interventions, pain, chronic pain, research methods, neuroscience, evidence based practice

## Introduction

Mechanistic research refers to investigating and measuring a health-related change resulting from an intervention (1). Such research typically requires a large dataset and highly controlled protocols, which has been challenging for music therapy researchers (2), especially for those who prioritize complex, individualized needs contextualized in systems that affect access to healthcare and impose traumas that compound patients' pain experiences. I will discuss these tensions and propose ways that mechanistic research into music and pain interventions can be clinically relevant for music therapists. This discussion grows in urgency as more patients seek treatment for pain associated with long COVID (3) and as researchers gain more understanding of the role of neuroplasticity in chronic pain (4), increasing demand for biopsychosocial pain interventions such as music therapy. Only recently have researchers focused on identifying and validating cognitive mechanisms of pain relief using music (5). The body of research investigating neurological mechanisms on music interventions for pain focus on music listening rather than music therapy interventions; two studies investigating neurological responses to music therapy for pain involve case studies (6) or address lab-induced pain (7). Though these support at least two different ways music therapy can support analgesia (music as distraction vs. music as active coping), more evidence is needed.

Music therapists should collaborate with researchers on undertaking mechanistic studies on music interventions that will lead to more effective, accessible, and relevant supports for pain management. I will highlight several research methodologies and how each approach is particularly relevant to this cause. Humanizing, intersubjective research approaches have the potential to capture the most effective elements of music as an experiential intervention. Outcomes of such research will help practitioners refine interventions and increase access to effective, music-based pain relief.

## Guiding values for research about music therapy for pain

The following core values are among those particularly important to music therapists conducting research with chronic pain populations.

### Inclusive evidence-based practice

For centuries, Western medical hierarchies have valued reductionistic, mechanistic studies to demonstrate the effectiveness of interventions. In the context of music therapy for pain, we need more clarity regarding whether and what kind of music is a mediating (causal) or moderating (affecting strength and direction) variable in pain relief, and what other factors may lead to beneficial outcomes. Though many medical practitioners and scientists advocate for more rigorous and urgent investigation into music therapy and music interventions (8), such research is ethically and clinically difficult for music therapists (2). Systematic reviews are at the top of the Evidence Based Practice (EBP) hierarchy, whereas qualitative studies are lower [e.g., as cited in Melnyk, (9)]. Groups such as the Cochrane Collaboration (https://www.cochranelibrary.com) aim to synthesize evidence of interventions to inform practitioners' choice of intervention; due to its strong influence, in 2016 90% of the WHO guidelines contained Cochrane evidence (https://www.cochrane.org/news/use-cochrane-reviews-inform-who-guidelines). Though there is no current Cochrane review on music for pain, several Cochrane reviews address music therapy and music medicine interventions in pain and medical contexts, each advocating for more robust clinical evidence (10–13). Magee and Stewart discuss how inclusion criteria for Cochrane reviews are narrowly defined, often excluding studies containing relevant qualitative data (2). Though considered less informative in the evidence hierarchy, these qualitative datasets have valid implications for treatment efficacy. Music therapists regularly witness patients' subjective responses within the music-based therapeutic relationship (14). Therefore, music therapists often advocate for a broader conceptualization of EBP, including rigorous qualitative and mixed methods research (15).

### Ecological validity

Given the limitations of standardized intervention delivery in a relational modality such as music therapy, researchers increasingly strive for ecological validity–designing research in naturalistic settings, and using individualized treatment approaches in the context of a therapeutic relationship. Holleman et al. argue that researchers should explain their rationale for such designs, defining the design's “naturality” and “complexity”, and recognizing the design's limitations (16). Data collection, the music experience, and the relationship with the therapist are all affected by the environment, personal experience and situatedness, and therapeutic intention (14). These factors are particularly important to address when investigating the fluid and subjective phenomenon of pain: clinicians want the freedom to exercise clinical decision-making as much as possible to replicate real-world experiences. Accordingly, research participants would experience individualized treatment in the context of a clinical relationship, rather than in a standardized delivery designed for a lab setting. Ecological validity must be a major consideration for research in this area, given the complexity of patients' pain experiences.

### Social justice

Westernized healthcare has often disenfranchised pain patients, particularly women and minorities (17, 18). Many such patients seek alternative means of pain support because of practitioners' lack of understanding of their pain experiences or lack of access to effective care. Where available and accessible, music therapy has been an option for such patients. Future researchers should assess whether new and refined interventions are feasible and can be made accessible for patients who have historically been marginalized from effective pain treatment, and they must intentionally study the impacts of systemic marginalization on the pain experience–including neurobiological effects. Researchers should integrate such findings with research on the effects of event-related and repeated trauma on the CNS, including to what degree symptoms of “catastrophizing” and “anxiety” (4, 8) are related to trauma and pain response, and understanding how different music interventions could address limbic system overactivation. Such work could link neurological biomarkers to cognitive mechanisms of music interventions for pain (5).

## Research approaches

Music therapist researchers may choose several approaches to accommodate these core values of inclusive EBP, ecological validity, and social justice.

### Flexible RCT protocols

Approaches permitting treatment individualization within a standard protocol are perhaps highest in the medical EBP hierarchy. Few such studies involve music therapy targeting pain in individuals, though these do not report outcomes on pain measures (19, 20) or the results are not yet published (21). Examples in other contexts include clinical improvisation for depression (22, 23) and autism (24) and customized songs for pre-term infants and their caregivers (25). A neuroimaging study (26) resulting from Erkkilä et al., (22) found that twelve weeks of individualized music improvisation led to resting-state brain changes in depressed participants, perhaps related to affective expression. This result could relate to chronic pain patients whose symptoms correspond with dysregulated mood and trauma history, a potential avenue for future investigation.

A flexible RCT design utilizing neuroimaging could explicate the unique role of music therapy vs. other relational interventions, clarifying music therapy's clinical significance. If music therapy interventions lead to identifiable activation/physiological responses, these could inform effective treatments. For example, should the different neural responses to contrasting music interventions observed in Hauck et al. (7) and Hunt et al. (6) prove to be robust in a flexible RCT, then clinicians would be better informed in selecting a music listening vs. an entrainment intervention for a given patient experiencing pain. Biomarkers may predict treatment responses to pain, and determine criteria for indications/contraindications for specific interventions, perhaps identifying the role of neuroplasticity in chronic pain and the degree to which music interventions can affect pain perception and neural organization, or how to best support patients with persistent neuropathic pain resulting from viral infections such as COVID-19. Furthermore, such biomarkers can be validating to patients who have had no explanation for their pain—affirming their experiences while supporting the benefits of nonpharmacological interventions focusing on biopsychosocialspiritual domains.

### Mixed methods

Many music therapists are familiar with the potential of mixed methods designs to help explain the nuances of music interventions; Bradt et al. (27) give an overview of such designs particularly useful for music therapy research. Despite their great potential, there are still few mixed methods studies, perhaps due to their complexity and challenges in publishing outcomes (28). Examples in music and pain research include the mixed method intervention design (29) employed by Bradt et al. (30) and Low et al. (31). In both studies, researchers embedded semistructured interviews within an RCT. The qualitative responses highlighted the limitations of standardized instruments for the target population and also helped refine understanding of the mechanisms of change. For example, in Bradt et al. (30), focus group participants shared how the quality of life scale lacked relevance to their lived experience due to its assumptions about participants' socioeconomic and social status. Participants in both Bradt et al. (30) and Low et al. (31) also explained how they were unable to report all their perceived benefits of the intervention *via* the standardized measures, and how unexpected outcomes related to beneficial behaviors that improved participants' quality of life. Mixed methods approaches continue to evolve according to researchers' questions and needs. Neurophenomenology and social neuroscience approaches also integrate quantitative and qualitative data to address questions that are highly relevant for investigating music interventions for pain.

### Neurophenomenology

Neurophenomenology, initially developed by Varela (32), seeks to undertake neurobiological investigations of subjectivity and consciousness. The approach has evolved from the very focused investigation of brief mental and sensory tasks (e.g., 33) to include an integrated investigation of the biological and subjective experience of a guided music and imagery session (34). Given the wide range of foci and data, there is a continuum of sequencing and integrating phenomenological data with neuroimaging, summarized in Berkovich-Ohana et al. (35). Generally, practitioner-researchers would identify the phenomenological focus of the clinical intervention and determine whether to examine neuroimaging data and phenomenological investigation simultaneously or in different sequences. These approaches would yield rich information regarding both the pain experience and different kinds of music experiences–whether receptive or active, provided by a music therapist or music medicine practitioner, and at any level of practice, perhaps using levels described by Dileo (36) including Distraction/Refocusing, Supportive, Cathartic/Expressive, Existential, and/or Transformational. Thus neurophenomenology offers flexible approaches to integrating biomechanistic information with patients' subjective pain experiences in the context of music interventions.

### Social neuroscience

Like neurophenomenology, social neuroscience approaches seek to preserve the ecological validity of the target phenomena, while focusing on experiences where the therapeutic relationship is the primary mechanism of change (37). This approach is best suited for interventions where the musical relationship is primary (“music *as* therapy” rather than “music *in* therapy”; 38), and where researchers seek to investigate ongoing music experiences *in vivo* rather than discrete, decontextualized stimuli. Previous studies of this kind have investigated the relationship between multiple participants' physiological signals using EEG and/or ECG (hyperscanning) to determine patterns of physiological synchronization aligned with moments of interest (MOI; as mutually identified by research participants) during a therapy session. This approach aligns musical interaction with physiological changes as they occur over time, providing a structure to investigate mechanisms of change (37). Several examples include analysis of EEG and ECG and clinical improvisation between client-therapist dyads in stroke rehabilitation (15), EEG and music-evoked imagery between a participant-therapist dyad in a psychotherapy session (39), EEG and active music therapy between children and their observing parents (40), and EEG and active music therapy between a participant and clinician and the participant's observing parent (41). Understanding how individual brains relate to interactive, relational therapies can help shape the therapeutic approach. Thus, Tucek et al. (15) and Kang et al. (41) propose that research in this area could seek to optimize intervention strategies for individual patients, perhaps by automating MOI detection in neural signals based on neural and subjective data, indicating when the dyad experiences the most effective moments of “engagement, insight, emotional intensity, and regulation” (15, p. 19). This approach can work with nuances of patients' pain experiences, which fluctuate in response to many factors. It is well suited for pain interventions such as Entrainment (42)/Music Imaginative Pain Treatment (43) which harnesses the musical relationship between client and therapist to support pain relief. For example, an investigator would examine the physiological synchronization between participant and therapist during therapist- and participant-identified MOIs during the improvised pain and healing music, and integrate these analyses with the participant's subjective post-session pain reports.

## Discussion

Given the complexity of researching music interventions for pain, no wonder music therapists may resist a narrow focus on biomechanistic RCT research. As well as investigating physiological responses to an intervention, mechanisms may also be realized across biopsychosocialspiritual domains. For example, research showing self-efficacy as a benefit of music therapy for pain (31) and as a consequence of a sequence of cognitive, affective, sensory, and phenomenological experiencing of music listening for pain relief (5) demonstrate the interrelatedness of these domains. This calls for an increased understanding of the nested situatedness of individuals, groups, communities, and systems in which clinicians and their participants live and receive care. To accomplish such wide-ranging investigations, the field needs more collaboration among diverse research groups, each with expertise in particular approaches, driven by their mission to investigate, develop, and refine feasible and acceptable music-based pain relief. One example of such a collaboration is the International Association for Music and Medicine (IAMM) Special Interest Group on Music Therapy and Chronic Pain (44) which keeps abreast of current research, explores methodological and theoretical concerns to address in future studies, and identifies research priorities–all while centering patients' and stakeholders’ voices.

Whereas research approaches striving for ecological validity can help with such questions, we still must consider the larger social context and health infrastructures in which these interventions are embedded. Public health experts, ethnographers, and social scientists could help investigate these systemic mechanisms *via* participatory action research (PAR) projects involving minoritized groups and general medicine practitioners to develop effective implementation/adaptation of music interventions for pain (e.g., PAR design in Ref. 45). Such approaches can explicate the medical systems and sociocultural barriers to music-based pain care in a given context. In a PAR project, community advocates and other stakeholders would guide and give feedback to researchers, helping them to refine the intervention to best meet that community's needs.

Accordingly, ethical, practical, and relevant research into the mechanisms of music therapy interventions for pain is possible. Such research requires transdisciplinary clinical and research collaboration with careful attention to contextual layers, where experienced music therapists can guide teams to effectively identify and navigate participants' unique clinical situations. Research teams should also situate their work in the larger body of research and their communities to enhance our collective understanding.
